# Evaluation of the mosquitocidal efficacy of fluralaner, a potential candidate for drug based vector control

**DOI:** 10.1038/s41598-024-56053-x

**Published:** 2024-03-07

**Authors:** Harish Kumar Shah, Vaishnavi Srinivasan, Shakila Venkatesan, Vijayakumar Balakrishnan, Sadanandane Candasamy, Nisha Mathew, Ashwani Kumar, Vijesh Sreedhar Kuttiatt

**Affiliations:** 1 ICMR-Vector Control Research Centre, Puducherry, 605 006 India; 2 Present Address: Saveetha Institute of Medical and Technical Sciences, Saveetha University, Thandalam, Kanchipuram, 602 105 India

**Keywords:** Fluralaner, Endectocide, Drug based vector control, Mosquitocidal effect, Life history characteristics, Entomology, Pharmacology

## Abstract

Vector control is a key intervention against mosquito borne diseases. However, conventional methods have several limitations and alternate strategies are in urgent need. Vector control with endectocides such as ivermectin is emerging as a novel strategy. The short half-life of ivermectin is a limiting factor for its application as a mass therapy tool for vector control. Isoxazoline compounds like fluralaner, a class of veterinary acaricides with long half-life hold promise as an alternative. However, information about their mosquitocidal effect is limited. We explored the efficacy of fluralaner against laboratory reared vector mosquitoes—*Aedes aegypti, Anopheles stephensi*, and, *Culex quinquefasciatus*. 24 h post-blood feeding, fluralaner showed a significant mosquitocidal effect with LC_50_ values in the range of 24.04–49.82 ng/mL for the three different mosquito species tested. Effects on life history characteristics (fecundity, egg hatch success, etc.) were also observed and significant effects were noted at drug concentrations of 20, 25 and 45 ng/mL for *Ae. aegypti*, *An. stephensi*, and, *Cx. quinquefasciatus* respectively. At higher drug concentration of 250 ng/mL, significant mortality was observed within 1–2 h of post blood feeding. Potent mosquitocidal effect coupled with its long half-life makes fluralaner an excellent candidate for drug based vector control strategies.

## Introduction

Vector borne diseases such as malaria, lymphatic filariasis, dengue and Zika virus pose significant public health threats globally^1^. Effective vaccines or specific treatment are not available for many of these diseases and vector control is the only option for prevention and control^2^. However, conventional vector control methods have several limitations and there is an urgent need for alternative control strategies. With the discovery of the potent mosquitocidal effect of the anti-parasitic drug ivermectin, vector control with endectocides is emerging as an alternative strategy^3,4^. This approach involves mass administration of a systemic insecticidal drug to animal or human population aimed at killing the blood feeding arthropods. Ivermectin is now being explored as a mass drug administration (MDA) tool in humans for malaria control in many settings^5–8^. As we progress towards malaria elimination, due to the extensive use of LLIN (Long Lasting Insecticidal Nets) and IRS (Indoor Residual Spraying), mosquitoes’ feeding behavior may change and they may prefer to feed on animals rather than humans^9^. Hence mass therapy of domestic animals with ivermectin is also suggested^10–13^. However, one major drawback for application of ivermectin for vector control is its short half-life (2.07 ± 0.71 days)^14^. This would necessitate its very frequent administration to human or animal population which may limit its utility in mass therapy. But drugs with shorter half-life tend to be safer and slow release formulation could bypass the need of frequent administration^13^.

Alternative endectocides with longer half-life which are proved to be safe or having only minimal side effects in animals and humans are highly desirable. Isoxazoline compounds like fluralaner (Bravecto®) and afoxolaner (NexGard®) used as acaricides in veterinary practice have an unusually long half-life (14.27 ± 2.53 days)^15,16^. After a single oral administration (50 mg/kg) of fluralaner in dogs, maximum plasma concentrations (C_max_) of 5419 ± 2086 ng/mL have been achieved within 24 h and detectable plasma levels were found up to 112 days post administration^15,17^. The target binding sites of fluralaner are similar to those of ivermectin, although their mechanisms of action differ. Fluralaner acts as a potent inhibitor or antagonist of γ-amino butyric acid (GABA) and l-glutamate-gated chloride channels, inducing spastic paralysis in insects. Notably, it binds to GABA channels more efficiently. In contrast, ivermectin binds more efficiently to glutamate channels and serves as an agonist, resulting in flaccid paralysis in insects^18^. Isoxazoline compounds may have minimal or no effect on the vertebrate nervous system^15^. Dogs treated with fluralaner and then exposed to mosquitoes have shown a significant reduction in the survival and fecundity of the fed mosquitoes for up to 13 weeks post-treatment^19^. Furthermore, fluralaner has demonstrated effectiveness in controlling the transmission of *Dirofilaria immitis* (heartworm) by killing vector mosquitoes that fed on dogs treated with the drug^20^.

With its excellent safety profile in animals, long half-life and the potent acaricidal effect, fluralaner can be an alternative choice for drug based vector control strategies. However, information on its mosquitocidal effect is very limited^21^. A recent study from USA (United States of America) reported its significant lethal effect in different mosquito vector species such as *Anopheles stephensi* (Liston, 1901), *Anopheles gambiae* Kisumu, *Anopheles gambiae* Tiassalé (Gile, 1902), *Aedes aegypti* New Orleans, *Aedes aegypti* Cayman (Linnaeus, 1762) and *Culex pipiens* (Linnaeus, 1758) (Diptera: Culicidae) species^21^.

In the present study, the adulticidal effect of fluralaner was studied against three vector mosquito species from India: (1) *Aedes aegypti*, the primary vector of dengue, (2) *Anopheles stephensi*, the vector of urban malaria, and (3) *Culex quinquefasciatus* (Say, 1823) (Diptera: Culicidae), the vector of lymphatic filariasis under laboratory conditions. In addition, the effect of fluralaner on fecundity, egg hatch success, immature development and adult emergence success were investigated on mosquitoes fed with different concentrations of the drug.

## Results

### Estimated LC_50_ value of fluralaner at different time points

Based on the preliminary results of initial bioassays, experiments were performed with narrow range drug concentrations (with three technical replicates), as described in methodology section for each of the species and the 24 h LC_50_ (95% CI) value was estimated. The results are summarized in (Table 1). Since the upper limit for 95% CI of 24 h LC_50_ was exceeding the maximum dosage used for *Ae. aegypti* and *An. stephensi*, the mortality data was subjected to cox-regression analysis to compare the survivorship or risk of death between different treatment and control group.

**Table 1:** Lethal concentration values.

Species	Post blood feeding time (in hours)	Slope (± SE)	Chi^2^ goodness of fit (P- value)	LC_50_ (95% CI)	Heterogeneity factor
*Aedes aegypti*	24	0.11 (0.004)	3.13 (0.08)	24.04 (14.74–45.27)	3.13
*Anopheles stephensi*	24	0.12 (0.005)	2.40 (0.12)	33.22 (24.30–51.56)	2.40
*Culex quinquefasciatus*	24	0.17 (0.01)	0.11 (0.90)	49.82 (49.35–50.29)	0.11

Probit regression analysis: SE, standard error; CI, confidence interval; LC, lethal concentration.

### Cox-regression analysis

#### *Ae. aegypti*

**Figure 1:**
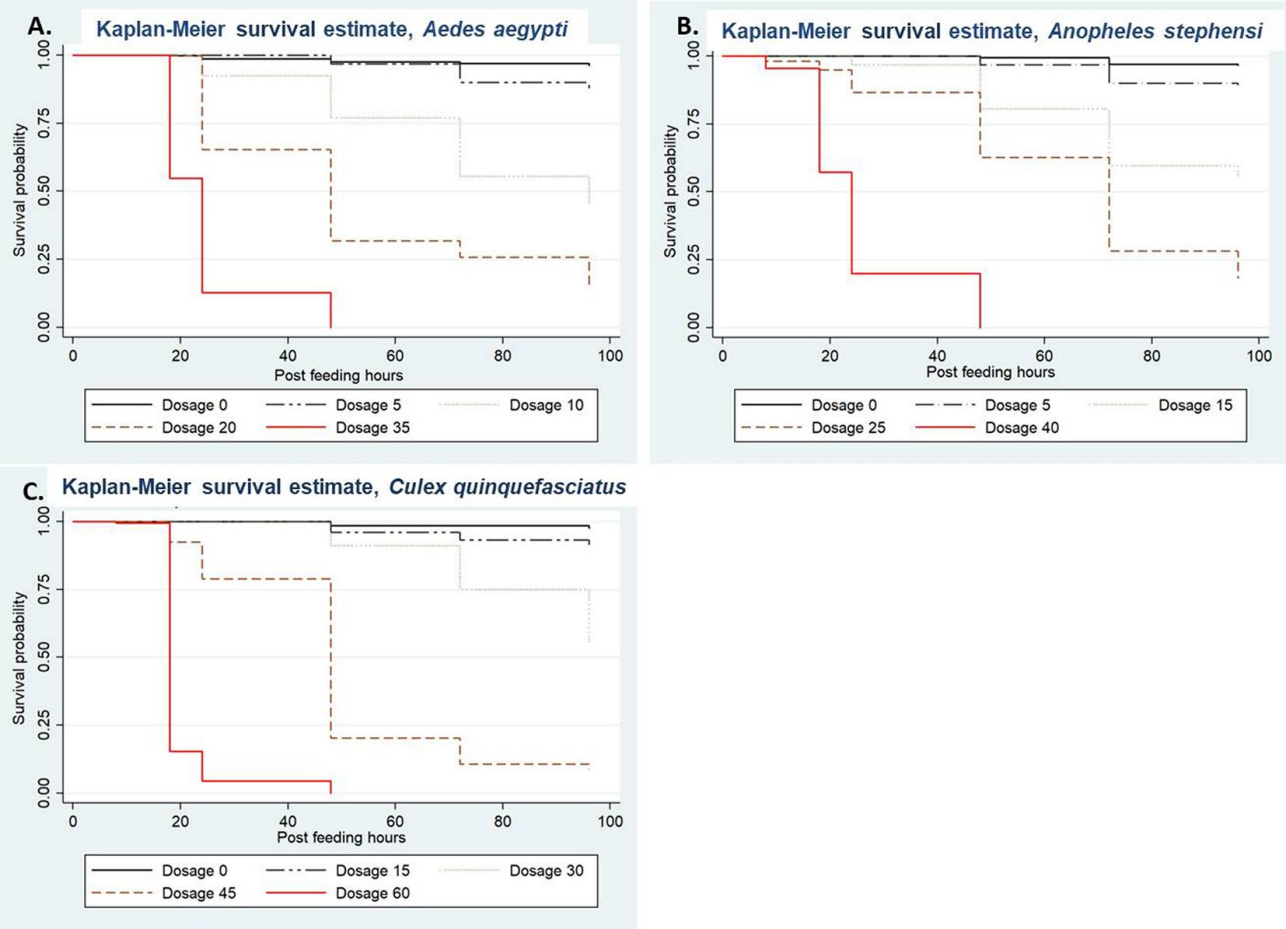
Effect of fluralaner on survivorship of treated mosquitoes in comparison with control (dosage 0 ng/mL). (**A**) *Aedes aegypti*. (**B**) *Anopheles stephensi*. (**C**) *Culex quinquefasciatus.*

The risk of death was significantly higher in dosage 35 (HR = 150.45 (95% CI: 94.76–238.87), P-value < 0.001) when compared to control i.e., dosage 0 (Table 2) (Fig. 1A).

**Table 2:** Effect of fluralaner on survivorship of *Aedes aegypti*, *Anopheles stephensi* and *Culex quinquefasciatus*.

Dose	Median survival duration (in hours)	Cox-regression analysis	
		HR (95% CI)	P-value
*Aedes aegypti*			
0 (Control)	–	1.00	–
5 ng/mL	–	3.45 (2.07–5.74)	< 0.001
10 ng/mL	96	17.19 (10.86–27.22)	< 0.001
20 ng/mL	48	40.61 (25.77–64.01)	< 0.001
35 ng/mL	24	150.45 (94.76–238.87)	< 0.001
*Anopheles stephensi*			
0 (Control)	–	1.00	–
5 ng/mL	–	3.48 (2.02–5.97)	< 0.001
15 ng/mL	–	17.21 (10.63–27.88)	< 0.001
25 ng/mL	72	39.72 (24.73–63.80)	< 0.001
40 ng/mL	24	231.94 (142.68–377.05)	< 0.001
*Culex quinquefasciatus*			
0 (Control)	–	1.00	–
15 ng/mL	–	3.72 (1.99–7.00)	< 0.001
30 ng/mL	–	20.82 (11.88–36.49)	< 0.001
45 ng/mL	48	91.53 (52.45–159.75)	< 0.001
60 ng/mL	18	516.41 (292.07–913.07)	< 0.001

Cox-regression analysis: HR, hazard ratio; CI, confidence interval.

#### *An. stephensi*

The risk of death was significantly higher in dosage 40 (HR = 231.94 (95% CI: 142.68–377.05), P-value < 0.001) when compared to control i.e., dosage 0 (Table 2) (Fig. 1B).

#### *Cx. quinquefasciatus*

The risk of death was significantly higher in dosage 60 (HR = 516.41 (95% CI: 292.07–913.07), P-value < 0.001) when compared to control i.e., dosage 0 (Table 2) (Fig. 1C).

### Effects on life history characteristics of mosquitoes fed with fluralaner

### Fecundity

The overall fecundity (average number of eggs laid per female mosquito) of *Ae. aegypti, An. stephensi* and *Cx. quinquefasciatus* females treated with fluralaner at different drug concentrations are given in (Table 3). The mean fecundity differed significantly between treated and control groups at drug concentrations nearing 24 h LC_50_ value for all the three species tested (P-value < 0.05) (Table 3). At higher drug concentration, dead adults with half of the abdomen filled with eggs were found in the oviposition cups in case of *An. stephensi and Cx. quinquefasciatus.* In the case of *Cx. quinquefasciatus* distorted egg rafts were found in the oviposition cups i.e., many eggs were scattered.

**Table 3:** Fecundity success.

Mosquito species	Fluralaner (ng/mL)	Number of eggs laid per mosquito Mean (SD)				Mean difference	P-value
		n	Treated	n	Control		
*Aedes aegypti*	5	48	95.10 (1.70)	49	97.70 (0.60)	2.60	0.018
	10	39	91.90 (1.30)	49	96.70 (0.60)	4.80	0.012
	20	16	78.70 (1.00)	48	96.70 (1.10)	18.00	0.011
*Anopheles stephensi*	5	48	88.30 (0.50)	50	90.30 (0.60)	2.00	0.007
	15	40	84.20 (1.50)	50	90.00 (0.00)	5.80	0.011
	25	31	72.00 (2.10)	50	89.30 (0.60)	17.30	0.012
*Culex quinquefasciatus*	15	47	124.20 (2.60)	49	136.00 (3.60)	11.80	0.012
	30	40	120.30 (2.10)	49	134.30 (2.50)	14.00	0.011
	45	7	117.30 (3.00)	49	135.30 (1.50)	18.00	0.012

n = number of mosquitoes kept for fecundity.

### Egg hatch success

The egg hatch success of *Ae. aegypti, An. stephensi* and *Cx. quinquefasciatus* mosquitoes treated with fluralaner at different drug concentrations are given in Table 4. The mean egg hatch success differed significantly between the treated and control groups (P-value < 0.05) at higher drug concentrations, compared to the effect observed at lower concentrations (Table 4).

**Table 4:** Egg hatch success.

Mosquito species	Fluralaner (ng/mL)	Number of eggs hatched Mean (SD)				Mean difference	P-value
		n	Treated	n	Control		
*Aedes aegypti*	5	500	403.67 (4.74)	500	414.00 (6.24)	10.33	0.026
	10	500	395.67 (5.29)	500	419.67 (4.04)	24.00	0.013
	20	500	386.00 (5.17)	500	416.00 (3.60)	30.00	0.012
*Anopheles stephensi*	5	500	435.22 (6.96)	500	456.33 (4.51)	21.11	0.013
	15	500	420.22 (7.05)	500	452.67 (3.05)	32.44	0.013
	25	500	408.89 (4.94)	500	453.33 (12.86)	44.44	0.013
*Culex quinquefasciatus*	15	517	426.22 (9.97)	533	462.66 (8.14)	36.44	0.013
	30	524	425.11 (11.88)	515	446.00 (18.68)	20.89	0.060
	45	518	417.22 (4.82)	531	457.33 (24.68)	40.11	0.012

n = number of eggs kept for hatching.

### Immature development

The overall immature development of *Ae. aegypti, An. stephensi* and *Cx. quinquefasciatus* mosquitoes treated with fluralaner at different drug concentrations are given in Table 5. The mean immature development varied significantly between control and treatment groups at higher drug concentrations tested: for *Ae. aegypti*20 ng/mL; for *An. stephensi*25 ng/mL; for *Cx. quinquefasciatus*45 ng/mL (P-value < 0.05 for all species).

**Table 5:** Immature survival.

Mosquito species	Fluralaner (ng/mL)	Number of pupae developed Mean (SD)				Mean difference	P-value
		n	Treated	n	Control		
*Aedes aegypti*	5	100	98.80 (0.70)	100	99.70 (0.60)	0.90	0.07
	10	100	97.40 (1.10)	100	99.00 (1.00)	1.60	0.07
	20	100	91.90 (2.60)	100	99.00 (0.00)	7.10	0.01
*Anopheles stephensi*	5	100	99.10 (0.80)	100	100.00 (0.00)	0.90	0.07
	15	100	97.80 (1.40)	100	99.00 (0.00)	1.20	0.11
	25	100	96.40 (1.30)	100	98.70 (0.60)	2.20	0.01
*Culex quinquefasciatus*	15	100	98.20 (0.70)	100	99.30 (1.10)	1.10	0.11
	30	100	93.80 (1.90)	100	99.30 (0.60)	5.60	0.01
	45	100	89.20 (3.00)	100	99.30 (0.60)	10.10	0.01

n = number of larvae kept for pupal development.

### Adult emergence success

**Figure 2:**
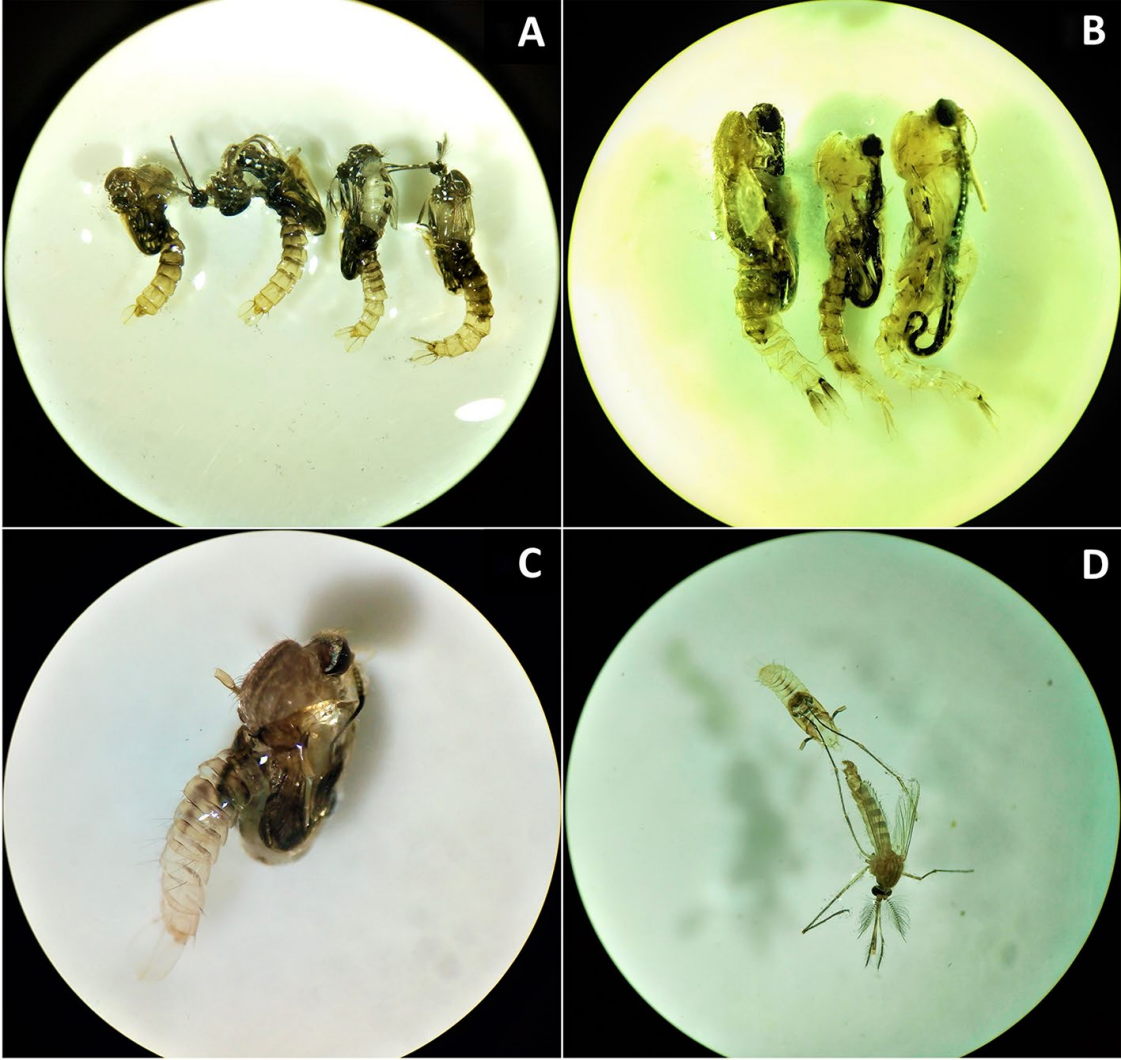
(**A**) Partially emerged dead adult *Ae. aegypti* mosquitoes. (**B**) Partially emerged dead adult *An. stephensi* mosquitoes. (**C**) Partially emerged dead adult *Cx. quinquefasciatus* mosquitoes. (**D**) Adult *Cx. quinquefasciatus* mosquito with legs attached to pupal exuviae.

The overall adult emergence success of *Ae. aegypti, An. stephensi* and *Cx. quinquefasciatus* mosquitoes exposed to fluralaner at different drug concentrations are given in Table 6. Certain morphological abnormalities in a few drug exposed mosquitoes at concentration nearing 24 h LC_50_ (20 ng/mL, 25 ng/mL, 45 ng/mL for *Ae. Aegypti, An. stephensi,* and *Cx. quinquefasciatus* respectively) were observed, such as partially emerged adults and emerged adult mosquitoes with their legs attached to the pupal exuviae (Fig. 2A–D).

**Table 6:** Adult emergence success.

Mosquito species	Fluralaner (ng/mL)	Number of adults emerged Mean(SD)				Mean difference	P-value
		n	Treated	n	Control		
*Aedes aegypti*	5	50	49.56 (0.53)	50	50.00 (0.00)	0.44	0.17
	10	50	49.22 (0.67)	50	49.67 (0.58)	0.44	0.30
	20	50	48.44 (0.88)	50	50.00 (0.00)	1.56	0.02
*Anopheles stephensi*	5	50	49.44 (0.53)	50	50.00 (0.00)	0.56	0.10
	15	50	48.00 (0.71)	50	49.67 (0.58)	1.67	0.01
	25	50	46.78 (1.30)	50	48.67 (0.58)	1.89	0.04
*Culex quinquefasciatus*	15	50	49.56 (0.53)	50	50.00 (0.00)	0.44	0.17
	30	50	48.78 (0.44)	50	49.67 (0.58)	0.89	0.02
	45	50	47.67 (1.32)	50	49.67 (0.58)	2.00	0.02

n = number of pupae kept for adult emergence.

### Estimation of mortality over time

**Figure 3:**
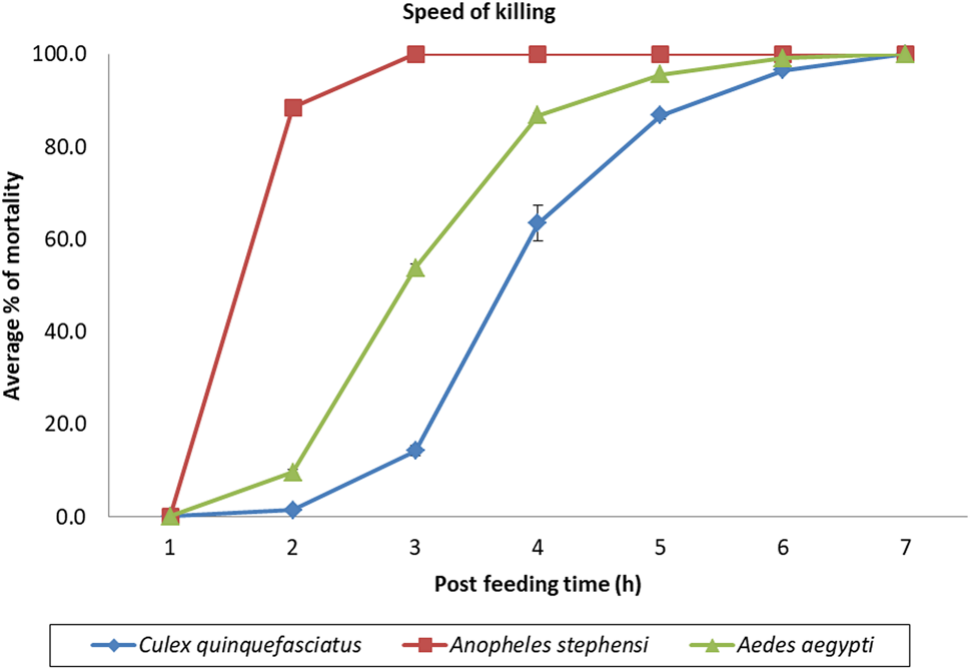
Mean percent mortality at different time point observed with three species of vector mosquitoes treated with fluralaner at a concentration of 250 ng/mL.

Estimation of mortality at different time points on feeding with different concentrations of the drug for all the three species is depicted in supplementary graphs 1–3. Speed of killing was also studied at a concentration 4–5 folds higher (250 ng/mL) (Fig. 3). When treated with this concentration, *An. stephensi* showed 100.00% mortality at 3 h of post blood feeding. In the case of *Ae. aegypti*, 99.00% mortality was recorded at 6 h of post blood feeding. For *Cx. quinquefasciatus,* it took 7 h to produce 100.00% mortality post blood feeding with fluralaner. The LT_50_ values for all the three species at given concentration is provided in Table 7.

**Table 7:** Lethal time (LT_50_) values at 250 ng/mL.

Species	Fluralaner (ng/mL)	LT_50_ (h) (95% CI)
*Aedes aegypti*	250	2.65 (2.58–2.70)
*Anopheles stephensi*	250	1.63 (1.55–1.70)
*Culex quinquefasciatus*	250	3.76 (3.65–3.87)

## Discussion

An ideal endectocide for administration as a mass therapy tool for vector control should have potent mosquitocidal effect along with long half-life property so that the drug effect lasts for weeks to months after one-time drug administration. In the present study, oral treatment with fluralaner resulted in significant mosquitocidal effect against the three vector species studied, with the estimated 24 h LC_50_ values ranging from 24.04 ng/mL to 49.82 ng/mL. The current study is the first attempt that examined the effect of fluralaner against *Cx. quinquefasciatus*.

Mosquitocidal effect of fluralaner observed in the current study was similar to that of in an earlier study, which reported 24 h LC_50_ values of 18.48–51.52 ng/mL^21^. The 24 h LC_50_ value of fluralaner observed in the earlier study as well as in the current study was lower than that reported for ivermectin (LC_50_: 44.68–49.40 ng/mL)^22^. After a single oral administration of fluralaner at 50 mg/kg body weight in dogs, 100 ng/mL concentration of fluralaner was maintained in the blood for a period of 3 months^15^. Mosquitoes fed on dog treated with fluralaner were reported to have significant reduction in their survival and fecundity for up to 13 weeks post-treatment^19^. It is noteworthy that the blood level concentration of fluralaner observed in dogs for three months is well above the 24 h LC_50_ values (24.04–49.82 ng/mL) of fluralaner observed against three species in the current study.

Fluralaner affected the fecundity, egg hatch success, immature development and adult emergence success in all the three species when they were exposed to drug concentrations nearing 24 h LC_50_ values of 24.04 ng/mL for *Ae. aegypti*, 33.22 ng/mL for *An. stephensi* and 49.82 ng/mL for *Cx. quinquefasciatus* with significant P value. Although these differences were statistically significant, this may not have any importance under field conditions. There was no effect or only minimal effect at lower drug concentrations. In contrast, a significant decrease was observed in the fecundity, egg hatch, larval and pupal development in the groups exposed to a sublethal dose of fluralaner in *An. aquasalis* ^23^. Similar finding was reported for ivermectin which showed significant effect on fecundity and egg hatch success at sub lethal concentrations—LC_5_ value 18.28 ng/mL^22^. It is curious that fluralaner’s anti-fecundity activity is relatively lesser to that of ivermectin, even though their mortality effects are similar post blood feeding in different mosquito species. One possible reason for this effect could be that ivermectin and isoxazoline compounds may have different mechanism of action regarding the anti-fecundity effect. Further research may be desirable in understanding this differential effect. Though fluralaner did not cause any visible impact on the development of the pupae into adults, certain morphological abnormalities like partially emerged adults and emerged adults with their legs attached to the pupal exuviae were observed. In an earlier study, fluralaner has been shown to affect the adult emergence success in *Spodoptera litura* Fabricius (Lepidoptera: Noctuidae), even at sub-lethal dose of LC_15_ morphological abnormalities like notched wings were observed in the adults^24^.

Studies on susceptible and resistant strains of horn and house flies have shown high level of mortality at lower concentration with fluralaner when compared with imidacloprid^25^. In a similar study on fipronil and pyrethroid resistant bed bugs, fluralaner outperformed ivermectin with a high mortality rate up to 28 days of post-treatment^26^.

In the current study, complete mortality was observed within 7 h of post blood feeding at 250 ng/mL drug concentration in all the three species tested. Blood concentration much above this level is expected during the initial weeks in animals treated with fluralaner. This is a notable advantage over ivermectin which takes 72 h to produce 70.00–100.00% morality in mosquitoes at a dose of 93 ng/mL in animal experiments^14,27,28^ and also this plasma level lasts for less than one day only.

Currently, ivermectin mass therapy is being investigated in peri-domestic animals and humans in field studies for its endectocidal effect and as a complementary tool for malaria vector control^10,11,29^. Fluralaner, with its comparable mosquitocidal effect to ivermectin coupled with the long half–life may be a good alternative to ivermectin. Additionally, fluralaner was found to be effective in controlling *Dirofilaria immitis* (heartworm) transmission by killing the vector mosquitoes that fed on drug treated dogs^20^.

With several emerging and remerging vector borne and zoonotic diseases, there is a great interest in ‘One-Health’ efforts being enforced round the globe. This would be particularly relevant in Africa and Asia where humans and animals live in close proximity and the drug based vector control strategy holds promise in such settings^30^. Moreover, it would be useful in insecticide resistance management plans due to its distinct binding site from that of the known modulators of ionotropic GABA receptors^22,31^. Though fluralaner appears to be a promising candidate for vector control, there are some limitations that are to be resolved before proceeding further. High cost of fluralaner is a barrier. Another issue is the safety aspects of fluralaner in human beings. It is not licensed for use in human beings. However, no adverse events were reported when fluralaner and afoxolaner were used for treating scabies in human clinical trials^32,33^.

There are some limitations of the current study. We used chicken blood, not human blood for carrying out the efficacy trials for convenience. Another limitation is that we have carried out the study only in laboratory maintained colonies and not on field caught mosquitoes. The number of survived mosquitoes assessed for life history characteristics were not comparable between treatment and control groups. The lab obtained results may not be exactly replicable in field conditions. Another limitation is that we did not perform direct blood feeding experiments with mosquitoes fed upon animals treated with fluralaner. This experiment is necessary to understand the effect of the drug on mosquitoes in the context of its metabolism in the treated animals.

In conclusion, our study showed significant oral toxic effect of fluralaner in adult vector mosquitoes with additional effect on life history characteristics (reduced fecundity, egg hatch success, larval development and adult emergence). Fluralaner may be a suitable candidate for future drug based mosquito control strategies. It is required to carry out future safety studies of fluralaner in human beings.

## Methods

### Extraction of fluralaner from the commercial tablet

Fluralaner was extracted from the commercially available tablets for veterinary use (Bravecto®-purchased from local veterinary drug shop; manufactured by Merck Animal Health, Madison, Vienna, Austria) using the method described by Miglianico et al.^21^. The tablet was crushed into fine powder using a pestle and mortar and then dissolved in a solvent prepared by mixing dichloromethane and methanol in a 1:1 ratio. The whole mixture was then agitated at room temperature for 1 h with a magnetic stirrer and filtered using Whatman® grade 1 filter paper (Sigma-Aldrich, St. Louis, MO, USA). The filtrate was left overnight for evaporation and the concentrated final product was obtained. Stock solution (5 mg/mL) was prepared in dimethyl sulfoxide (DMSO) (HiMedia, Mumbai, India) and aliquots were frozen at − 20 °C. Prior to each experiment, aliquots were dissolved in phosphate buffer saline (PBS) (Sigma-Aldrich, St. Louis, MO, USA) and added to heparinized chicken blood to achieve the desired final drug concentrations required for the mosquito blood feeding experiments.

### Vector mosquitoes and colony maintenance

Laboratory reared mosquito species of *Ae. aegypti*, *An. stephensi* and *Cx. quinquefasciatus* were obtained from the rearing and colonization facility at the Indian Council of Medical Research—Vector Control Research Centre (ICMR-VCRC), Puducherry, India. These strains were originally collected from the Union Territory of Puducherry, India and the colonies have been maintained in the insectary since 1975. *Cx. quinquefasciatus* were collected in 1975 and *Ae. aegypti* and *An. stephensi* were collected in 1976. They were maintained at a constant temperature of 27 ± 2 °C and relative humidity of 80 ± 10% in one-foot Barraud cages (30 cm L × 30 cm W × 30 cm H) and provided with 10% sucrose solution.

### Laboratory bioassays with fluralaner

To determine the activity range of fluralaner, initially, bioassays were performed to assess the mortality effect at 24 h using wide range concentrations (20–120 ng/mL). Based on the results of these experiments, a narrow range of four drug concentrations were selected for bioassays to determine LC_50_ (concentration resulting in 50% mortality after taking a blood meal) value at 24 h of post blood feeding. Drug concentrations of 5 ng/mL, 10 ng/mL, 20 ng/mL and 35 ng/mL were selected for the feeding experiments with *Ae aegypti;* 5 ng/mL, 15 ng/mL, 25 ng/mL and 40 ng/mL for *An. stephensi*; and 15 ng/mL, 30 ng/mL, 45 ng/mL and 60 ng/mL for *Cx. quinquefasciatus* respectively (two drug concentration above and below LC_50_ value of initial bioassays)*.*

Five days old, non-blood fed female mosquitoes from same batch were used for blood feeding experiments. Prior to blood feeding, cotton pads soaked with 10% sucrose solution were removed and the adult mosquitoes were allowed to starve at least for 12 h to improve the feeding rate. Five mL of heparinized chicken blood containing desired concentration of fluralaner was used for feeding the mosquitoes in the treatment group via artificial blood feeding apparatus made of glass fitted with blood mixing rotor and parafilm for about 60–90 min. Treatment group consists of four drug concentrations and each concentration constituted of three technical replicate cages (each with 50 females). 50 adult females from the same batch of mosquitoes fed on chicken blood with only solvent were used as controls. After blood feeding, mosquitoes (fully engorged) were transferred to clean cages, provided with 10% sucrose solution and the mortality effect was observed at 4, 8, 18, 24, 48, 72 and 96 h of post feeding. 48 h (*Ae. aegypti*, *An. stephensi*) and 72 h (*Cx. quinquefasciatus*) of post blood feeding, survived mosquitoes were observed for fecundity. The experiment was repeated three times on different days using fresh batches of mosquitoes and freshly prepared fluralaner concentration as biological replicates.

In continuation to the bioassay experiments for mortality effect of fluralaner, life history characteristics of survived mosquitoes in each drug treatment groups were assessed in comparison to the control groups. Number of survived mosquitoes in each replicate cage varied with the species and also with the drug concentration.

### Effect of fluralaner on fecundity, egg hatch success, immature development and adult emergence success

For fecundity assessment of survived *Ae. aegypti* and *An. stephensi*, oviposition cups filled with 75–90 mL water and lined with filter paper were introduced into the cages at 48 h of post blood feeding. For *Cx. quinquefasciatus*, oviposition cups without filter paper were introduced into the cages at 72 h as it takes 3 days to become fully gravid. All the oviposition cups were placed in the center of the cage. To assess the effect of fluralaner in egg hatch success, eggs laid by survived mosquitoes in treatment group were compared with that of eggs laid by mosquitoes in the control group. For hatching experiment of *Ae. aegypti* and *An. stephensi*, five hundred eggs counted under microscope were floated in trays (45 cm L × 30 cm W × 10 cm H) filled with water using a fine tip brush. In case of *Cx. quinquefasciatus*, as eggs were laid in rafts, numbers were slightly more than five hundred. The rafts were carefully transferred to tray using brush without damaging it. Hundred first instar larvae hatched from eggs laid by survived mosquitoes were observed for development up to pupal stage by providing mosquito larval food [yeast (40%) and dog biscuit (60%)] once in 2 days and were compared with the control group. Rearing water was changed every 2 days. Daily mortality was recorded (if any) in both treatment and control group larvae. Similarly fifty pupae from survived mosquitoes were observed for adult emergence by transferring them into containers filled with 3/4^th^ water and covered with mosquito net having hole in middle plugged with cotton. The results were compared with the control group.

### Estimation of speed of killing at high concentration

We performed a set of experiments to assess the speed of killing of the mosquitoes when they were fed with a high concentration of fluralaner. In the initial weeks of drug administration in animals, the blood concentration is expected to be higher which may result in rapid killing of mosquitoes within hours of blood feeding. Consequently, a drug concentration level, specifically five times the highest LC_50_ value (49.82 ng/mL) obtained in the study, was selected to study the speed of killing. For this, 50 females in triplicates were fed at 250 ng/mL and were observed at hourly interval until complete (100%) mortality was noted in the treatment groups. The experiment was replicated three times on different days with fresh batch of mosquitoes.

### Statistical analysis

Mean and standard deviation value from three replicates of different concentration were taken to obtain the data, which was further used for various statistical analysis. Mortality data over time were subjected to Probit regression analysis to estimate LC_50_ and LT_50_ values. Mean (SD) and Frequency (%) were used to describe summary values. Mann Whitney U test was used to test the difference in fecundity, egg hatch success, immature survival and adult emergence success between the drug treated and control groups. P-value < 0.05 was considered as statistically significant. SPSS 16.0 was used for carrying out Probit analysis whereas, STATA 14.2 software for other statistical analyses.

### Supplementary Information

Supplementary Figures.

## Data Availability

The datasets generated and/or analyzed during the current study are available from the corresponding author on request.
