# Supplementary material for: Evaluation of the mosquitocidal efficacy of fluralaner, a potential candidate for drug based vector control
Source: Sci Rep. 2024 Mar 7;14:5628. doi: 10.1038/s41598-024-56053-x (PMC10920869; doi:10.1038/s41598-024-56053-x)

S1: Mean percent mortality observed with *Ae. aegypti* adults treated with different concentration of fluralaner at different time points

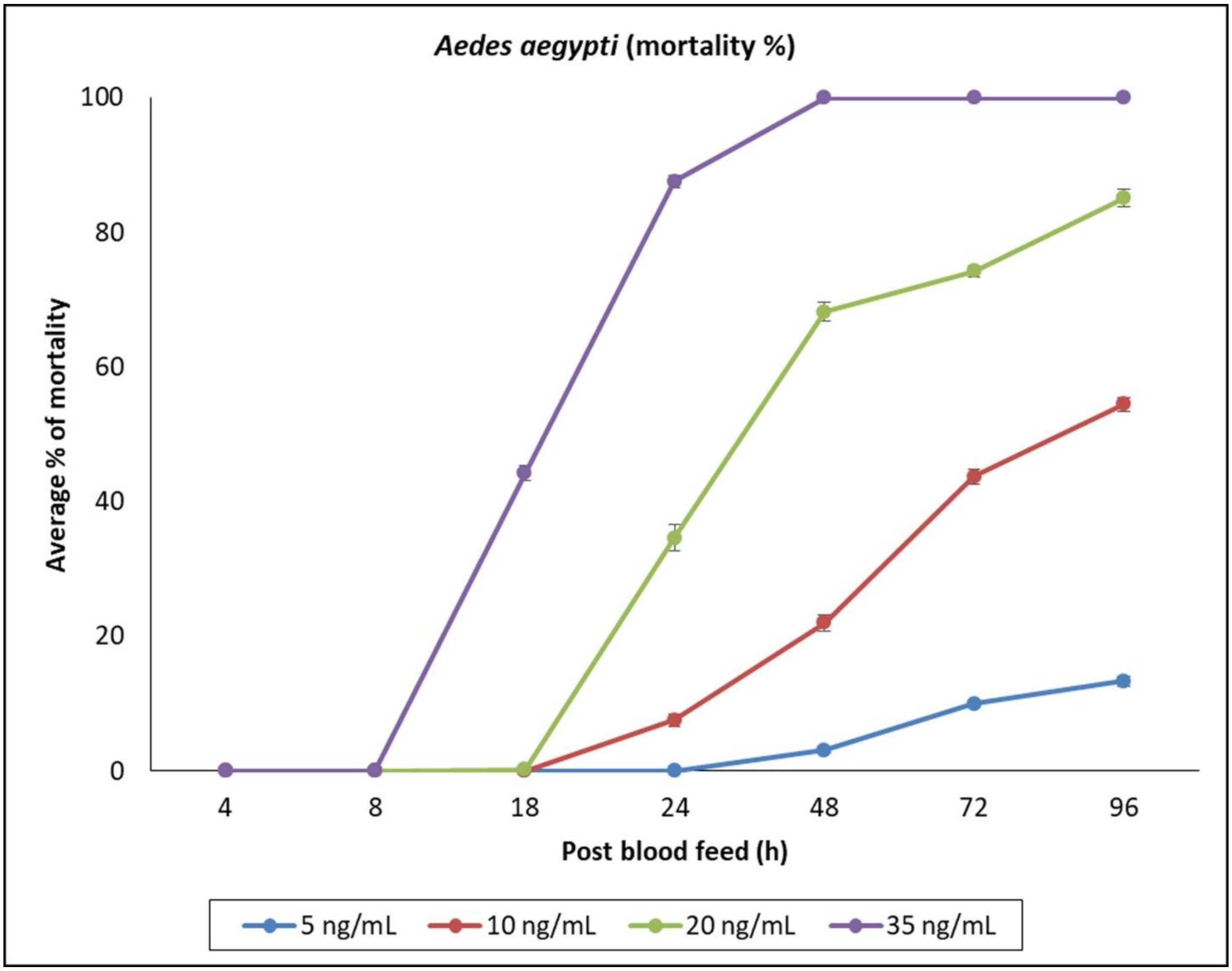

S2: Mean percent mortality observed with *An. stephensi* adults treated with different concentration of fluralaner at different time point

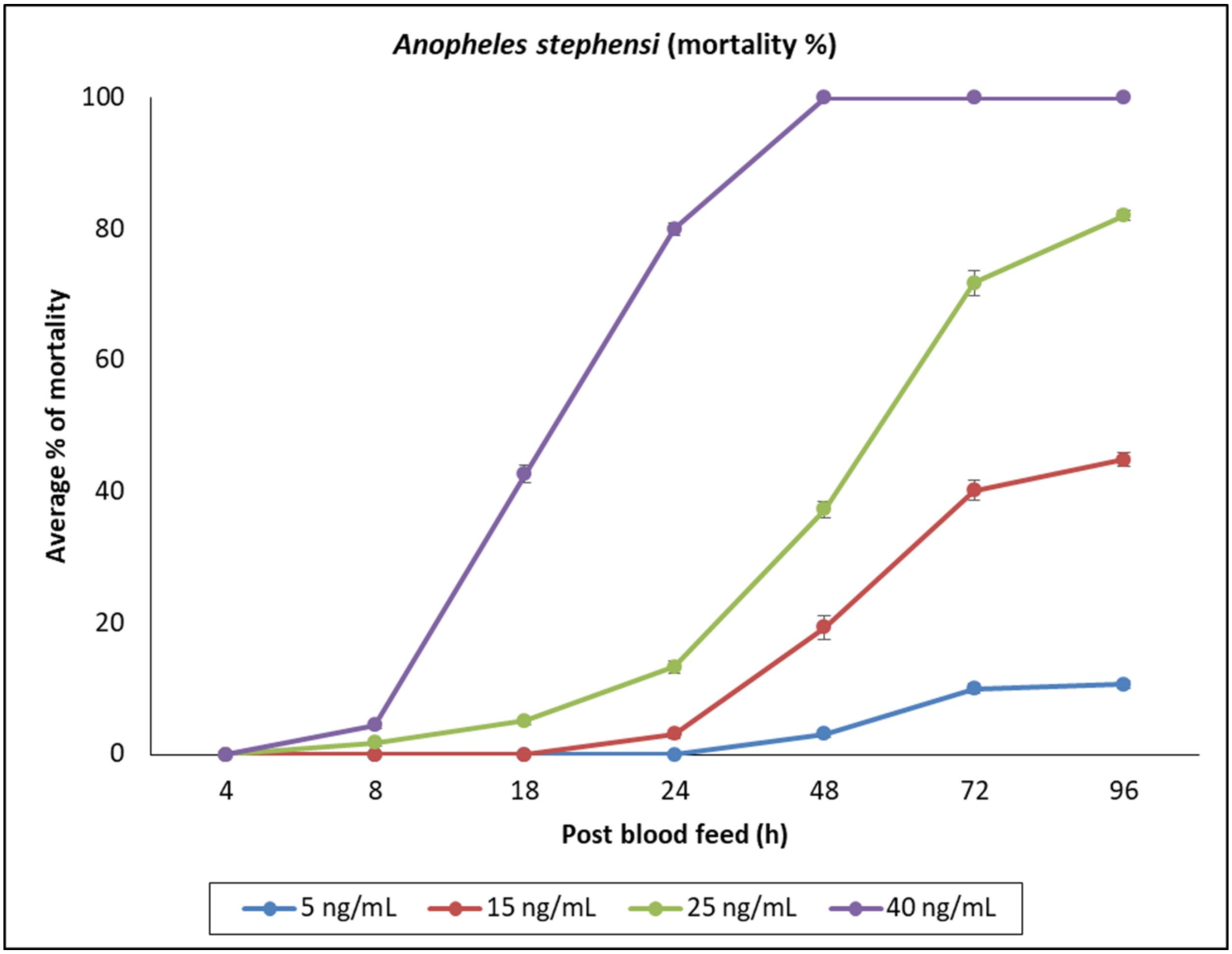

S3: Mean percent mortality observed with *Cx. quinquefasciatus* adults treated with different concentration of fluralaner at different time point

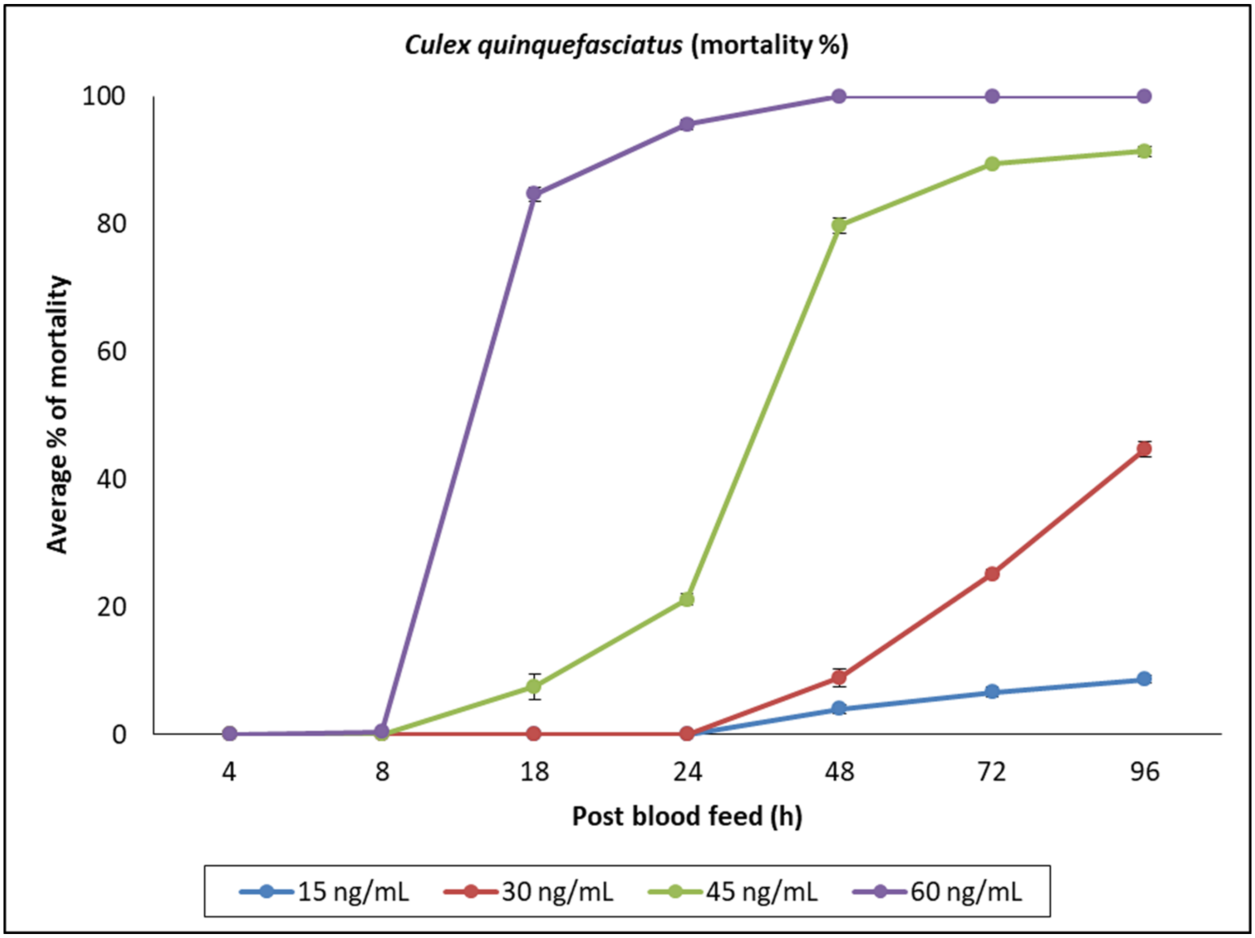

S4: Dose response curve for *Ae. aegypti* at 24 h post blood feeding

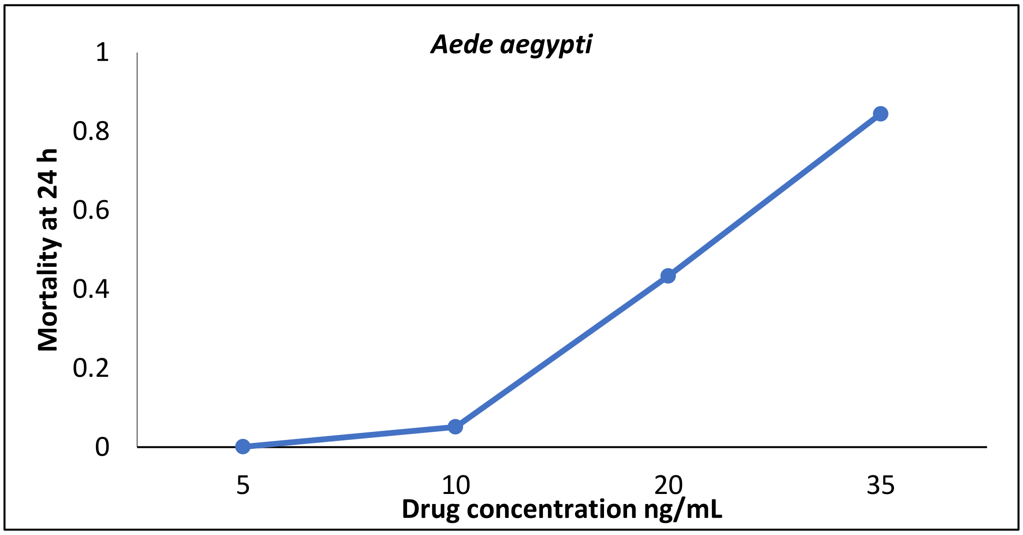

S5: Dose response curve for *An. stephensi* at 24 h post blood feeding

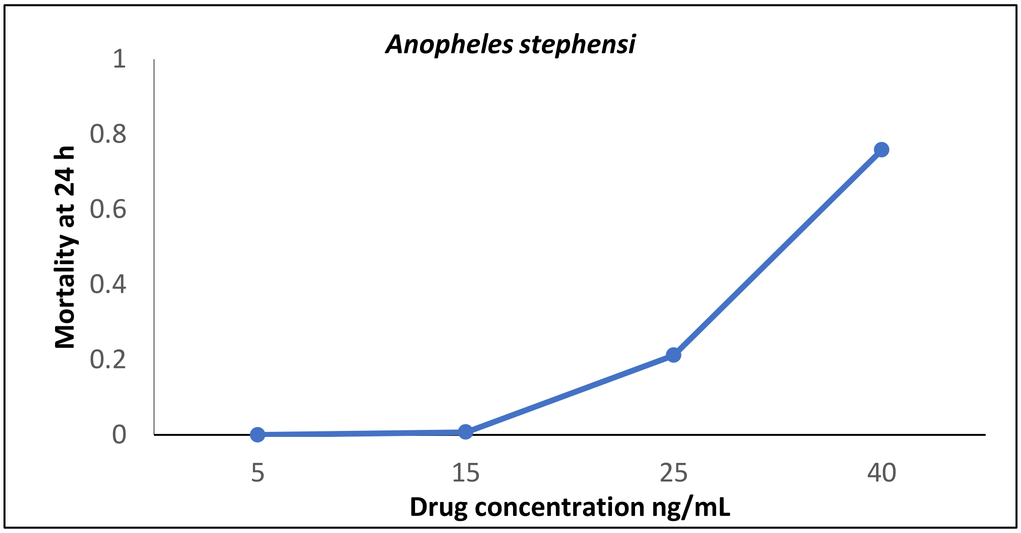

S6: Dose response curve for *Cx. quinquefasciatus* at 24 h post blood feeding

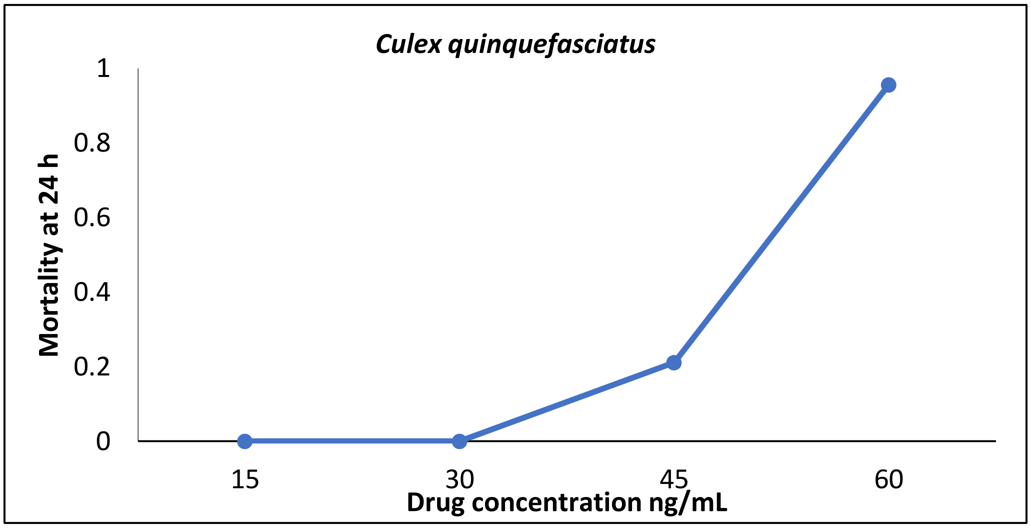

Supplement: Supplementary file 1 — Supplementary Figures. [file 41598_2024_56053_MOESM1_ESM.pdf]
